# Hunting dogs as sentinel animals for monitoring infections with *Trichinella* spp. in wildlife

**DOI:** 10.1186/s13071-016-1437-1

**Published:** 2016-03-16

**Authors:** Maria Angeles Gómez-Morales, Marco Selmi, Alessandra Ludovisi, Marco Amati, Eleonora Fiorentino, Lorenzo Breviglieri, Giovanni Poglayen, Edoardo Pozio

**Affiliations:** Department of Infectious, Parasitic and Immunomediated Diseases, Istituto Superiore di Sanità, Rome, Italy; U.F. Sanità Pubblica Veterinaria e Sicurezza Alimentare, ASL 2, Lucca, Italy; School of Agriculture and Veterinary Medicine, University of Bologna, Ozzano Emilia, Italy

**Keywords:** *Trichinella*, *Toxocara canis*, Helminths, Hunting dogs, Serology, ELISA, Western blot, Epidemiology, Wildlife

## Abstract

**Background:**

Nematode parasites of the genus *Trichinella* are important foodborne pathogens transmitted by ingestion of striated muscles harbouring infective larvae. Wild carnivorous and omnivorous animals are the most important reservoirs of these parasites. Hunting activities play an important role in *Trichinella* spp. epidemiology. The aim of the present work was to assess if serological detection of anti-*Trichinella* IgG in hunting dogs can be a tool to indirectly monitor *Trichinella* spp. infections in wildlife.

**Methods:**

An ELISA and a Western blot (Wb) were developed and validated. To validate the assays, serum samples were collected from 598 dogs considered to be *Trichinella*-free, 15 naturally infected dogs, and six experimentally infected foxes. Sera were tested by ELISA with *Trichinella spiralis* excretory/secretory antigens. The diagnostic sensitivity and specificity of ELISA were 100 % (95 % CI: 83.89–100 %) and 95.65 % (95 % CI: 93.69–97.14 %), respectively. Sera from *Trichinella*-infected dogs/foxes tested by Wb showed a three-band pattern ranging from 48 to 72 kDa. Since the prevalence of *Toxocara canis* is very high in dogs, the specificity of the ELISA and Wb was further assessed by testing sera for anti-*T. canis* IgG using *T. canis* excretory/secretory antigens. No cross-reactivity was observed. To evaluate the test’s reliability in the field, serum samples were collected from wild boar hunting dogs from Central Italy where *Trichinella britovi* was circulating among wildlife.

**Results:**

Out of 384 hunting dog sera, 189 (49.2 %) tested positive by ELISA and of these, 56 (29.6 %) tested positive by Wb, showing an overall prevalence of 14.6 % (56/384) in the wild boar hunting dog population of the investigated area. The serological prevalence in hunting dogs was significantly (*P* < 0.001) associated with the hunting district’s altitude. This is in agreement with previous investigations, which had shown that the prevalence of *T. britovi* in wildlife was higher in mountainous areas than in lowland areas of Italy.

**Conclusion:**

The results suggest that the circulation of *Trichinella* spp. among wildlife can be monitored by testing sera from hunting dogs, which could act as sentinel animals of *Trichinella* spp. circulation in wildlife.

## Background

Nematodes of the genus *Trichinella* are zoonotic parasites that circulate among wildlife of all continents but Antarctica. However, when humans fail in the proper management of domestic animals and wildlife, infections with *Trichinella* spp. can be transferred from the sylvatic to the domestic environment, favouring transmission to humans [1]. Hunting activities can play an important role in the epidemiology of *Trichinella* spp., increasing the spread of game carcasses and their offal and scraps, which can be infected with larvae of *Trichinella* spp. in the striated muscle tissues, in both the sylvatic and domestic environments [2]. Consequently, the risk of infection for humans by the consumption of raw meat and meat-derived products from both domestic and game animal species can increase [3].

The wild boar (*Sus scrofa*) is one of the most important game animals in Europe, Asia and other continents including North and South America, where this species has been introduced for hunting activities [4, 5]. A common way of hunting wild boar is by dogs, which drive out the wild boar and push it towards hunters.

Carnivores of the family Canidae (e.g. jackal, red fox, raccoon dog, wolf) are important natural reservoir hosts of most species of *Trichinella* (i.e. *T. spiralis*, *T. nativa*, *T. britovi*, *T. pseudospiralis*, *T. murrelli*, *T. nelsoni* and *Trichinella* sp. T9) [6]. The owned and stray dogs have also been found to be frequently infected with *T. spiralis*, *T. nativa*, *T. britovi*, *T. murrelli* and *T. nelsoni* in many countries of the world due to their scavenger behavior [7–13].

The collection of a muscle biopsy in the search of larvae of *Trichinella* spp. in dogs is causing stress to the animal; furthermore, it is invasive and expensive. It follows that no muscle tissue can be easily sampled from dogs and tested to search for larvae of *Trichinella* spp.

The aim of the present work was to assess if the serological detection of anti-*Trichinella* IgG in hunting dogs can be considered a useful tool to monitor the circulation of these zoonotic nematodes among wild animals present in the hunting area. To this end, an ELISA for the detection of anti-*Trichinella* IgG in dog serum samples was developed and validated. Moreover, the *Trichinella*-specific antigens recognised by serum samples from *Trichinella* spp.-infected dogs were identified by Western blot (Wb) to define a distinctive pattern of *Trichinella* spp. infection in these sera. Finally, serum samples of hunting dogs from an area where *Trichinella britovi* was known to circulate in wildlife were tested using the validated tests. The results suggest that the circulation of *Trichinella* spp. among wildlife can be monitored by testing sera from hunting dogs, which can thereby act as sentinel animals for these foodborne pathogens.

## Methods

### Study design

**Fig. 1:**
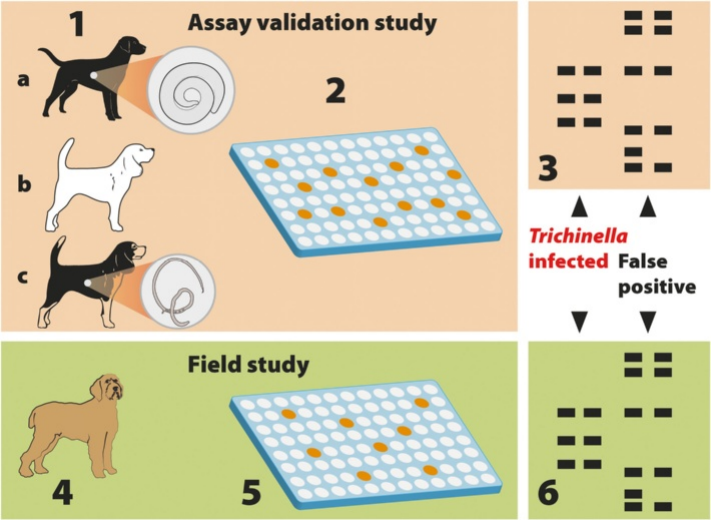
Study design. Assay validation study: 1. Serum samples were collected from *Trichinella* spp.-infected dogs and foxes (*n* = 21) (a), *Trichinella*-free dogs (*n* = 523) (b), and from *Trichinella*-free dogs (*n* = 75), which were infected with ancylostomatid nematodes, and/or *Diphylidium caninum*, and/or *Toxocara canis*, or *Dirofilaria immitis* (c). 2. Serum samples were tested by ELISA using excretory/secretory *Trichinella spiralis* muscle larva antigens (T_ESA). 3. ELISA-positive sera were tested by Western blot using T_ESA. Field study: 4. Serum samples were collected from wild boar hunting dogs (*n* = 384). 5. Sera were tested by the validated ELISA. 6. ELISA-positive sera were tested by the validated Western blot using T_ESA to distinguish sera of *Trichinella*-infected dogs from sera of false-positive dogs

For the validation of the assays (ELISA and Wb) to detect anti-*Trichinella* IgG in dog sera, serum samples were collected from the following groups of animals: (i) naturally *Trichinella*-infected dogs (8 from Serbia and 7 from Hungary) with larvae of *Trichinella* spp. detected in their muscles by artificial digestion, positive controls (*n* = 15); (ii) experimentally *Trichinella*-infected foxes with larvae of *Trichinella* spp. detected in their muscles by artificial digestion [14], positive controls (*n* = 6); (iii) dogs considered to be *Trichinella*-free based on their rearing conditions, negative controls (*n* = 523); and (iv) dogs infected with other helminths but *Trichinella*-free, for the evaluation of cross-reactions (*n* = 75) (Table 1; Fig. 1). Specifically these helminth (≠ *Trichinella*)-infected dogs were: 62 from the Brindisi province (Apulia region, southern Italy), an area where *Trichinella* spp. have never been documented in both wild and domestic susceptible animals [15]; these dogs were naturally infected with *Dipylidium caninum* and/or ancylostomatid nematodes and their serum samples tested negative for *Trichinella* by Wb; four stray dogs from Serbia (*n* = 3) and Hungary (*n* = 1), which tested negative for *Trichinella* infection by artificial digestion; and nine owned dogs infected with *Dirofilaria immitis* from the Emilia Romagna Region (northern Italy), which had tested negative for *Trichinella* by ELISA.

**Table 1:** Dog serum samples tested by ELISA and Western blot to detect anti-*Trichinella* spp. IgG and by ELISA to detect anti-*Toxocara canis* IgG

Animal origin	*Trichinella* spp.		*Toxocara canis*
	ELISA positive/tested (%)	Western blot positive/ELISA positive (%)	ELISA positive/tested (%)
Assay validation study			
*Trichinella* spp. positive controls			
Stray dogs^a^	8/8^a^	8/8	8/8
Farm dogs^b^	7/7	7/7	7/7
Laboratory silver foxes^c^	6/6	6/6	6/6
*Trichinella* spp. negative controls			
Laboratory dogs^d^	16/523 (3)	0/16	140/523 (26.7)
Helminth (≠ *Trichinella*) -infected dogs^e^	10/75 (13.3)	0/10	72/75 (96.0)
Total	47/619 (7.6)	21/47 (44.7)	233/619 (37.6)
Field study			
Hunting dogs^f^	189/384 (49.2)	56/189 (29.6)	372/384 (96.8)

^a^ Stray dogs from Serbia and ^b^ mongrel dogs from a farm in Hungary, which tested positive for larvae of *Trichinella* spp. by digestion; ^c^ foxes (*Vulpes vulpes*) experimentally infected with larvae of *T. spiralis*, kindly provided by Dr. Karsten Nöckler, Germany, and Dr. Rebecca K. Davidson, Norway; ^d^ 480 beagles and 43 of other breeds; ^e^ 62 dogs infected with ancylostomatid nematodes, and/or *Diphylidium caninum*, and/or *Toxocara canis*, from Apulia, an Italian region where *Trichinella* spp. have never been documented [15], four helminth (≠ *Trichinella*)-infected mongrel dogs from Serbia and Hungary which tested negative for *Trichinella* infection by artificial digestion, and nine owned dogs infected by *Dirofilaria immitis* from Emilia Romagna, an Italian region where *Trichinella* spp. circulate, which tested negative for *Trichinella* by ELISA; ^f^ wild boar hunting dogs of different breeds: shorthaired Italian hound, mongrel dog, grand bleu de Gascoigne, posavac hound, beagle, dachsbracke, Breton, and border collie

For the field study, 384 hunting dogs from the Lucca Province (Tuscany region, central Italy) were selected, because this hunting area has a large number of wild boar hunted per year (about 5000 heads per year), organised hunting teams, recording of hunted and *Trichinella* spp.-tested wild boar, and because of the occurrence of a trichinellosis outbreak caused by consumption of wild boar meat that involved hunters, their relatives and friends in 2012 [16] (Fig. 1).

Furthermore in order to evaluate the cross-reactivity, serum samples from all animals, both from the assay validation study and the field study, were tested for anti-*Toxocara canis* IgG, since it is one of the most prevalent nematodes in canids [17] (Table 1).

### Serum sample collection

With informed consent of the dog owners and adhering to a high standard of veterinary care, blood (3–5 ml) was collected from the cephalic vein of the foreleg of each dog, then it was allowed to clot and a serum sample was harvested, distributed in aliquots and frozen at -80 °C.

According to Italian legislation, regional territories are split into a number of hunting areas (ATC), and each ATC into several districts. Serum samples were collected from wild boar hunting dogs of ATC 11 (districts 11.1, 11.2, 11.12, 11.14, 11.15, 11.16, and 11.17) and ATC 12 (districts 12.1, 12.2, 12.3, 12.4, 12.5, 12.6, 12.7, 12.8, 12.9, 12.10, 12.11, and 12.13) of the Lucca Province with the help of the hunting associations in the course of 2013. The hunting dog owners were invited to gather their dogs at collection points for blood collection and to fill in a form with information on: age, sex, breed, length and type of hunting activity, number of dogs per hunting team, ATC and district of hunting, and microchip code. These dogs represented 32.3 % of the total number of wild boar hunting dogs hunting in the 19 districts.

### *Trichinella spiralis* and *Toxocara canis* excretory/secretory antigens

*Trichinella spiralis* excretory/secretory antigens (T_ESA) were prepared according to a previously published protocol [18]. *Toxocara canis* excretory/secretory antigens (Tox_ESA), kindly provided by Peter Deplazes (Zurich, Switzerland), were produced from L1 maintained in vitro [19].

### ELISA for *Trichinella* spp.

The dog and fox serum samples were first tested for the presence of anti-*Trichinella* IgG by ELISA using T_ESA. A protocol previously used for pig sera [20] was optimised and validated. Briefly, 96-well microtitre plates (Nunc-Immuno Plate Maxisorb, Roskilde, Denmark) were filled with 100 μl/well of T_ESA (5 μg/ml) in carbonate buffered saline pH 9.6 ± 0.2. After incubation at 37 °C for 1 h, plates were washed 3 times with an automatic plate washer (Dynex Technologies, Denkendorf, Germany) using washing solution (0.5 % Tween 20 in PBS pH 7.3 ± 0.2), then blocked by adding 200 μl/well of blocking solution (0.5 % BSA, 0.05 % Tween 20), and incubated at 37 °C for 1 h. After another washing, 100 μl/well of each 1/100 diluted serum sample were added in duplicate and the plates were incubated at 37 °C for 30 min. After washing again, 100 μl/well of 1/30,000 diluted horse radish peroxidase (HRPO) labeled anti-dog IgG (Kierkegaard and Perry Laboratories (KPL), Gaithersburg, MD, USA) [14] were added, and plates were incubated at 37 °C for 1 h. After a final wash, 100 μl/well of the substrate solution containing 3′,5,5′-tetramethylbenzidine and 0.02 % hydrogen peroxide in a citric acid buffer were added, and the plates were incubated at room temperature (RT) for 10 min. The reaction was stopped by adding 50 μl/well of 1 N HCl solution. The optical density (OD) was obtained by reading the reaction at 450 nm using an ELISA plate microtitre reader (Dynex Technologies, Chantilly, VA, USA). To validate the assay, the sensitivity, specificity, and the cut-off, were calculated following the recommendations of the World Organisation for Animal Health [21] and by receiver-operator characteristic (ROC) curve analysis [20, 22, 23]. The inter-assay variability was assessed by testing two negative and two positive serum samples in eight different work sessions and then calculating the coefficient of variation (CV).

### ELISA for *Toxocara canis*

All 619 dog serum samples from the assay validation study were tested by ELISA for the presence of anti-*T. canis* IgG to assess the specificity of the test according to Deplazes *et al.* [19] with some modifications. Briefly, plates (96-well microtiter plates) were coated with Tox_ESA diluted 1/10,000 in carbonate buffered saline (pH 9.6) for 1 h; then, the reaction was blocked with the blocking solution (2 % BSA, 0.05 % Tween 20) at 37 °C for 1 h, washed 3 times in PBS containing 0.05 % Tween 20, and 100 μL/well of dog serum diluted 1:20 were added in duplicate. Then the plates were incubated at 37 °C for 30 min. After 3 washings, 100 μL/well of 1:5000 diluted HRPO labeled anti-dog IgG antibody (KPL) were added, and the plates were further incubated at 37 °C for 1 h. The plates were washed before the addition of the substrate solution, and the above-reported procedure to detect anti-*Trichinella* IgG was then followed. For each serum sample, the OD was obtained by reading the reaction at 450 nm. On every ELISA plate, four negative control sera from laboratory dogs known to be free from nematodes were included. The cut-off value, 0.470, was calculated as the mean (+4 SD) of the OD values of 15 serum samples from dogs known to be free from intestinal nematodes [19].

### Western blot for *Trichinella* spp.

To confirm the specificity of the ELISA for *Trichinella* spp., serum samples that tested positive by ELISA were diluted 1:100 and then tested by Wb according to a previously published protocol [22]. Furthermore, to assess the quality of electrophoretic transfer in the gels, pre-stained molecular weight standards were used (Precision Plus Protein™ WesternC™ Standards, Bio-Rad, Hercules, CA, USA) in each run. The experiment was considered valid when all of the pre-stained protein standards (250, 150, 100, 75, 50, 37, 25 and 20 kD) were separated and transferred onto the nitrocellulose membrane and the relative mobility of each standard was within the standard range previously established by three independent experiments. The nitrocellulose filters were blocked with 5 % skimmed milk in 1× Tris-Borate saline-Tween (TBST, 50 mM Tris pH 8.0, 150 mM NaCL, 1 % Tween 20) at 4 °C overnight and washed three times with 1× TBST. Each nitrocellulose filter was cut into strips, each of which was then incubated with 1:100 dog serum with 3 % (w/v) skimmed milk (Sigma-Aldrich, Saint Louis, MO, USA) in 1× TBST at RT for 1 h. After washing 3 times with 1× TBST, the pre-stained protein standard strip was incubated with conjugated Precision ProteinTM Strep Tactin-HRP at 1:10,000 dilution, for 1 h. The other strips (previously incubated with the serum sample) were incubated with a 1:7000 dilution of goat anti-dog IgG conjugated HRP (KPL) for 1 h.

To reveal proteins with high efficiency, the LiteAblot® Plus chemiluminescence system (Euroclone, Pero, Milan, Italy) was added to the strips for 5 min. The proteins were then visualised on a ChemiDoc™ XRS System (Bio-Rad) and images were analysed using Image Lab™ software version 4.0 (Bio-Rad). The positivity/negativity of each serum sample was then determined by comparing the relative migration value (Rf) of each sample with that of the positive control on the same blot, and the corresponding MW was calculated by Image Lab™ software version 4.0 (Bio-Rad).

### Western blot for *Toxocara canis*

To compare the electrophoretic patterns of T_ESA and Tox_ESA and their reactivity with serum samples from *Trichinella* sp. or *T. canis* infected dogs, the procedure described below was followed. Tox_ESA were electrophoretically separated by SDS-PAGE on 10 % pre-cast NuPage Novex Bis–Tris Gels® (Invitrogen). Gels were stained with Coomassie blue G 250 (Bio-Rad) and visualised. For Wb, proteins were transferred to nitrocellulose (Bio-Rad) at RT for 1 h. A pre-stained standard low-range MW was used (104, 94, 51, 36, 28 and 19 kDa, Bio-Rad) in each run. Nitrocellulose filters were blocked with 5 % skimmed milk in 1× Tris-Borate Saline-Tween (TBST, 50 mM Tris, pH 8.0, 150 mM NaCl, 1 % Tween 20) at 4 °C overnight and washed three times with 1 % TBST. Each nitrocellulose strip was then incubated with 1:100 dog sera with 3 % w/v skimmed milk (Sigma-Aldrich) in 1 % TBST at RT for 1 h. After washing three times with 1 % TBST, the strips were incubated for 1 h with a 1:7000 dilution of goat anti-dog IgG conjugated with horseradish peroxidase HRPO (KPL). Reactive protein bands were revealed by 3,3-diaminobenzidine substrate (Sigma-Aldrich).

### Statistical analysis

To evaluate the sensitivity and specificity of the ELISA for *Trichinella* spp. a receiver-operator characteristic (ROC) curve analysis was carried out using EpiTools’ epidemiological calculators [23]. The Kappa coefficient was calculated as a statistical measure of agreement between the ELISA to detect anti-*Trichinella* IgG and the ELISA to detect anti-*Toxocara canis* IgG. This measure of agreement falls between 0, when the level of agreement is what would be expected by chance, and 1, when there is perfect agreement [23].

The statistical significance between the presence of anti-*Trichinella* spp. IgG in hunting dogs and the biological (age, sex, race), epidemiological (length and type of hunting activity, number of dogs per hunting team), and environmental (hunting district elevation) variables, was calculated using the software R [24]. The r.stat package was used to analyse exploratory statistics of the Digital Elevation Model raster of the study area and to calculate the mean elevation value of each hunting district [25]. The hypothesis of no association between groups was tested with Pearson’s Chi-squared test; a value of *P* < 0.05 was considered significant.

## Results

### ELISA validation

The diagnostic sensitivity and specificity of the ELISA to detect anti-*Trichinella* IgG in dog sera was 100 % (95 % CI: 83.89–100 %) and 95.65 % (95 % CI: 93.69–97.14 %) according to the ROC curve analysis, respectively, with 26 false positive serum samples, 16 from dogs considered to be *Trichinella*-free and ten from helminth (≠ *Trichinella*)-infected dogs (Table 1). The CV was 14 % and 12 % for the negative and positive serum samples, respectively. The cut-off value was 0.368 OD. The OD values of the six serum samples from *Trichinella*-positive foxes were higher than the cut-off value (0.368 OD) detected for dog sera.

**Fig. 2:**
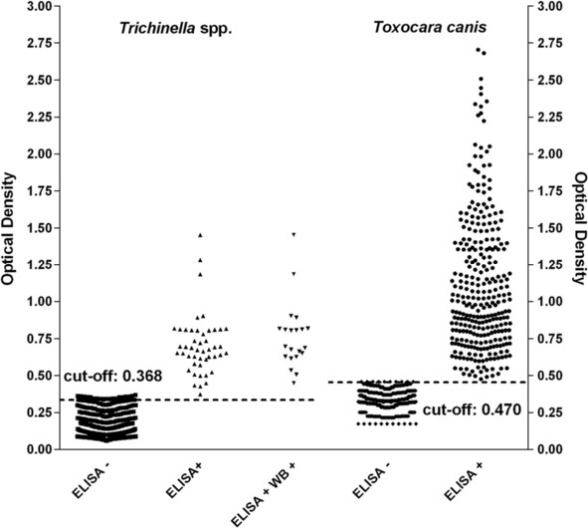
Assay validation study: optical density and cut-off values of dog/fox serum samples by ELISA. Serum samples from presumably *Trichinella* spp.-free (*n* = 598) and *Trichinella* spp.-infected (*n* = 21) dogs/foxes were tested by ELISA using *Trichinella spiralis* and *Toxocara canis* excretory/secretory antigens. Out of the 619 sera, 47 tested positive for *T. spiralis*; of these, 26 sera from presumably *Trichinella* spp.-free dogs tested negative by Western blot (Wb), and 21 sera from *Trichinella* spp.-infected dogs/foxes tested positive by Wb (see Table 1). Out of the 619 sera, 233 (37.6 %) tested positive by ELISA for *T. canis* (see Table 1)

The cut-off value of the ELISA to detect anti-*Toxocara* IgG in dog sera, was calculated to be 0.47 based on the mean + 4 SD of the OD values of the serum samples from 15 dogs, which tested negative for the presence of intestinal nematodes (Fig. 2).

Out of 619 serum samples, 47 (7.6 %) tested positive for both anti-*Trichinella* IgG and anti-*T. canis* IgG, and 233 (37.6 %) tested positive for anti-*T. canis* IgG (Table 1). A low Kappa coefficient (0.238) was determined.

### Western blot validation

**Fig. 3:**
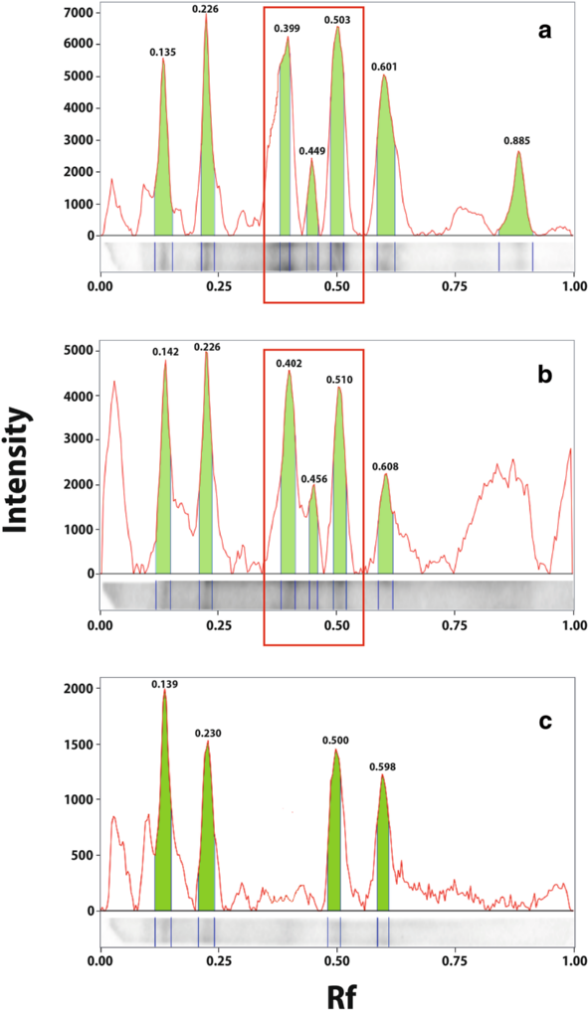
Assay validation study: *Trichinella spiralis* excretory/secretory antigens recognised by dog serum samples on Western blot. Signal intensities and relative migration values (Rf) of the proteins recognised by: **a**, a positive control serum from a naturally infected dog; **b**, serum sample from a hunting dog presumably infected by *Trichinella britovi*; **c**, an Ancylostomatidae-positive, *Trichinella* spp.-negative serum from a dog (false positive)

**Fig. 4:**
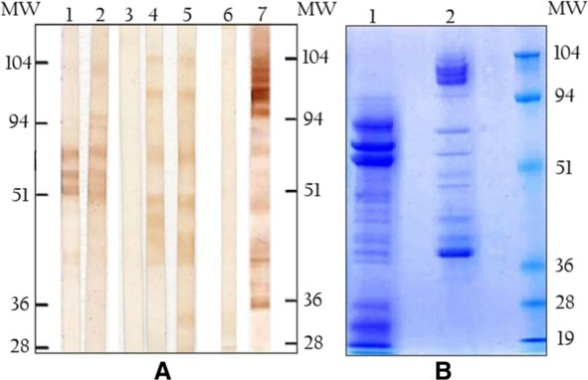
Assay validation study: western blot (Wb) and SDS-PAGE of excretory/secretory antigens from *Trichinella spiralis* (T_ESA) and from *Toxocara canis* (Tox_ESA). **a.** Lanes 1 and 2, T_ESA blotted with two serum samples from *T. spiralis* infected dogs. Lane 3, T_ESA blotted with a serum sample from a *Trichinella*-free dog. Lanes 4 and 5, T_ESA blotted with the serum samples from two *T. canis* infected dogs. Lane 6, Tox_ESA blotted with a serum sample from a *T. canis* uninfected dog; Lane 7, Tox_ESA blotted with a serum sample from a *T. canis* infected dog; Mw, molecular weight markers. **b**. Ten percent SDS-PAGE of T_ESA (Lane 1) and Tox_ESA (Lane 2) stained with Coomassie Blue; Mw, molecular weight markers

To define the *Trichinella* spp. protein pattern most frequently recognised by sera from *Trichinella* spp.-infected dogs, the 21 control positive dog/fox sera were tested three times by Wb. All sera reacted with a three-band pattern ranging in size from 48–72 kDa, which was consistent with patterns previously identified for human and pig serum samples [18]. This pattern was considered to define *Trichinella* spp. infection in dog sera. An experiment was considered valid when the Rf value of the proteins was within the range previously established by the three independent experiments for each positive control (first band from 0.480 to 0.517 mm; second band from 0.370 to 0.462 mm; and third band from 0.328 to 0.437 mm). The positivity/negativity of each dog serum sample was then determined by comparing the Rf value of each sample with the positive control on the same blot (Fig. 3a, b). The *T. spiralis* protein pattern of reactivity with sera from *Trichinella* spp.-infected dogs was unique and clearly different from that with sera from dogs infected with other helminths such as *Dipylidium caninum*, ancylostomatid nematodes and/or *T. canis* (Fig. 3c; Fig. 4a)*.* This is in agreement with the electrophoretic patterns of T_ESA and Tox_ ESA, which were different under Comassie blue staining (Fig. 4b). Moreover, serum samples from *T. canis*-infected dogs reacted by Wb mainly with the Tox_ ESA proteins with a molecular weight higher than 94 kDa. Other reactive bands were also observed between 51 and 94 kDa and between 36 and 51 kDa (Fig. 4a).

### Field study

**Fig. 5:**
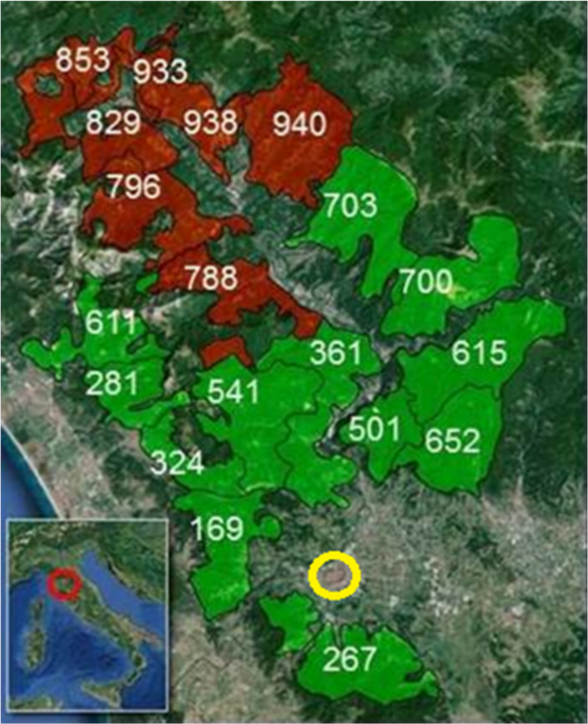
Field study: map of investigated hunting districts in the Lucca Province, Tuscany region, central Italy. The number on each hunting district is the altitude (in metres above the sea level). Brown areas indicate ATC 11 (average elevation 780 m asl; range 729–940 m); green areas indicate ATC 12 (average elevation 500 m asl; range 169–900 m). The yellow ring shows Lucca city

**Fig. 6:**
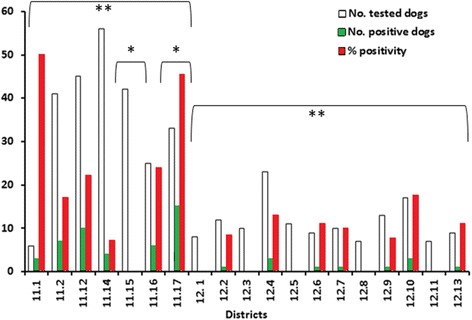
Field study: anti-*Trichinella* IgG prevalence in wild boar hunting dogs by hunting district in the Lucca province, Tuscany region, Central Italy. * *P* < 0.05; ** *P* < 0.001

Out of 384 serum samples from wild boar hunting dogs, 189 (49 %) tested positive by ELISA for *Trichinella* spp.; of these, 56 (29.6 %) tested positive by Wb, showing a prevalence of 14.5 % (56/384) in the hunting dog population of the investigated area (Table 1). The 56 dogs with anti-*Trichinella* IgG in their sera originated from 13 hunting districts (Fig. 5). The average serological prevalence was 19.6 % (49/249; range 7.1–50.0 %) in ATC 11, and 8.0 % (11/136; range 0–17.6 %) in ACT12, and this difference was statistically significant (*χ*^2^ = 15.611, *df* = 1, *P* < 0.0001); statistically significant differences were also observed among districts (*χ*^2^ = 8.075, *df* = 1, *P* = 0.043) (Fig. 6). No statistically significant differences were observed when positive dogs were stratified by age, sex, breed, length and type of hunting activity, or number of dogs per hunting team (data not shown). The average altitude of the ATC 11 districts (781 m above sea level, asl) was about 1/3 higher than the average altitude of the ATC 12 districts (500 m asl) (Fig. 5). By ELISA, 372 (96.8 %) serum samples from the hunting dogs tested positive for anti-*T. canis* IgG (Table 1); of these, 164 (42.7 %) serum samples also tested positive for anti-*Trichinella* IgG. The Kappa coefficient was poor (0.1191).

## Discussion

The role of domestic dogs as hosts of *Trichinella* spp. has been demonstrated experimentally since the 19th Century [26, 27]. In 1874, the first outbreak of human trichinellosis due to the consumption of dog meat was described in Germany [28]. In 1876, larvae of *Trichinella* spp. were detected in a naturally infected dog in Italy [29]. From 1897 to 1974, more than 75,000 dogs (about 68,000 in Europe; 3100 in North America; 490 in South America; 2750 in Asia; and 30 in Africa) were tested by direct assays in the course of 96 investigations, with a prevalence of *Trichinella* spp. ranging from 0 to 62 % [30].

According to the literature data from 1975 forwards, about 37,000 dogs were tested for *Trichinella* spp. by direct and indirect tests in different world regions, and about 21 % tested positive by digestion or by ELISA, most of which were from China [10, 12, 13, 31–35].

Up to now, even if an ELISA was used to detect anti-*Trichinella* IgG in dog sera, it was not properly validated and the Wb was rarely used as a confirmatory test. The first attempt to detect anti-*Trichinella* IgG in dog sera was done using antigens purified from *T. spiralis* larvae by Sephadex G-200 chromatography [36]. In this study, 66 serum samples were tested and a significantly higher detection rate was obtained by ELISA than by trichinoscopy. These *T. spiralis* antigens appeared not to cross-react with the sera of dogs infected with *Ancylostoma caninum* or *Taenia* spp.; however, data on validation was lacking [36]. Anti-*Trichinella* IgG were screened in dog sera from Greece by an ELISA using ES antigens using the serum of an experimentally infected dog as a positive control. A serological prevalence of 4.3 % was detected, but no information was reported on the cut-off value [31]. In Finland, an ELISA was used to test dog sera from a serum bank and serum samples from two experimentally infected raccoon dogs were used as positive controls. Dogs over 1 year of age had higher OD% than dogs less than 1 year of age [37]. However, the ELISA was not validated according to standard protocols. In another study, a commercial ELISA kit was used to test seven dog sera, but no information is available on the kit validation [32]. An ELISA was also used to test for anti-*Trichinella* IgG in serum and meat juice samples from foxes hunted in Belgium, but no confirmatory method was used to differentiate true positive from false positive serum samples [38]. More recently, anti-*Trichinella* IgG were screened by ELISA in dog sera from Vietnam. Positive sera were confirmed by Wb, finding a prevalence of 4 % in the investigated provinces; however, no information was provided on the test validation [35].

The ELISA to detect anti-*Trichinella* IgG developed and validated in this study showed good performance in terms of diagnostic sensitivity (100 %; 95 % CI: 83.89–100 %) and specificity (95.65 %; 95 % CI: 93.69–97.14 %). Thus, serum samples from the nine dogs infected with filarial worms, tested negative. Moreover, the Kappa coefficient between the ELISA for anti-*Trichinella* IgG and anti-*T. canis* IgG detected in laboratory (0.238) and in hunting (0.1191) dogs supports the poor correlation between the two tests, i.e. there is no cross-reaction between anti-*Trichinella* IgG and anti-*T. canis* IgG. Further, serum samples that had been tested as positive for anti-*Trichinella* IgG by ELISA were further tested by a validated highly sensitive Wb, which is based on the presence of a triple band pattern distinctive for *Trichinella* spp. infection. This pattern is different from that displayed by sera from dogs infected by ancylostomatid nematodes and *T. canis* (Figs. 3c, 4a).

The need to bind ELISA and Wb to detect the prevalence of anti-*Trichinella* IgG in a dog population, is highlighted by the difference between the percentage of ELISA positive sera and Wb positive sera, which shows that only 29.6 % of ELISA-positive sera were confirmed by Wb (Table 1). The serological prevalence detected in wild boar hunting dogs fits with epidemiological and environmental data. In fact, the average elevation of ACT 11 districts was 780 m asl (range 729–940 m); whereas the average elevation of ACT 12 districts was 500 m asl (range 169–900 m). In Italy, as well as in France and Spain, it has been observed that there is a relationship between the prevalence of *Trichinella* spp. in wildlife and elevation, i.e. the higher the prevalence, the higher the elevation [39]. In 2012, a wild boar infected with *T. britovi* was hunted in district 11.15 and the sausages made with its meat were the source of a trichinellosis outbreak infecting 34 people [16]. From 1998 to 2002, *T. britovi* was detected in three hunted foxes and one wolf found dead in this region, but no larvae of *Trichinella* spp. were detected in 129 foxes hunted in the region from 2004 to 2006 [40].

In only a few regions of the world, where wild boar are hunted, offal and scraps are disposed of properly. In most cases, after killing, wild boar carcasses are slaughtered on the field and offal and scraps are left on the ground. It follows that hunting dogs can ingest offal and scraps, which include striated muscles (frequently the whole diaphragm), i.e. the ecological niche of the larvae of *Trichinella* spp. In many areas of the world where hunting is practiced, hunting dogs have easy access to muscle samples of game including carnivore carcasses (e.g., foxes, mustelids, bears, walruses), left on the ground by hunters after skinning, or removing and discarding the entrails [41–46].

To monitor the circulation of *Trichinella* spp. in a region or a country, testing serum samples from hunting dogs represents a good compromise between the need for epidemiological information on the circulation of these zoonotic nematodes among wildlife and the difficulties in testing wild animals. Furthermore, since hunting dogs hunt in well-defined areas for a known period of time, a serological test combined with a questionnaire filled in by the owner, can provide information on the circulation of *Trichinella* spp. in a particular space and time, and regular testing of dog serum samples can provide information on the dynamics of these parasites in wildlife. However, since hunting dogs can also travel with their owners, this information should be reported in the questionnaire, together with information on dog’s feeding.

No information is available on anti-*Trichinella* IgG kinetics in dogs. In experimental studies, a larger immune response was detected in adult dogs than in puppies [47]. In foxes experimentally infected with 500–10,000 larvae, specific IgG were detected up to 72–100 weeks post-infection, which corresponds to the mean life expectancy of foxes in its natural habitat [48–50]. Likely, the hunting dogs studied in the present work acquired their infection with a smaller number of larvae of *Trichinella* spp., resulting in a lower and less persistent IgG response, as has been demonstrated in foxes experimentally infected with *T. nativa* [14]. The lack of a relationship between serological prevalence and the seniority hunting of a dog, or between serological prevalence and dog’s age, suggests that their immunological memory could be short.

## Conclusions

In this study, we have demonstrated that the presence of anti-*Trichinella* IgG can be detected in dog sera by a validated ELISA as screening test and by a validated Wb as confirmatory test, since sera from *Trichinella* spp.-infected dogs display a unique band pattern by Wb. In fact, no ambiguous Wb band patterns were observed when sera of *Trichinella* spp.-infected dogs and sera of helminth (≠ *Trichinella*)-infected dogs were compared.

Testing of hunting dog sera by validated assays could allow the circulation of *Trichinella* spp. in wildlife to be monitored, providing useful information for the risk assessment of game meat consumption in the areas investigated. Annual testing of hunting dogs for anti-*Trichinella* IgG could represent a way to maintain constant awareness by hunters to the risk of these zoonotic agents, achieving three beneficial objectives: (i) a reduction of carcasses, offal and scraps of game left on the ground; (ii) an increase in wild boar or other food animal carcasses tested for *Trichinella* spp. larvae by veterinary services; and (iii) education of the hunters, their families and friends regarding not consuming raw meat and meat-derived products from game animals. The collection of blood from dogs is easy and cheap and allows serum samples to be tested for a panel of antibodies against zoonotic (e.g. babesiosis, ehrlichiosis, leishmaniasis, leptospirosis, rickettsiosis) and non-zoonotic diseases (e.g. pseudorabies), and to monitor the health status of the dog, reducing the sampling costs. In conclusion, hunting dogs can act as sentinel animals for monitoring *Trichinella* spp. infections in wildlife.
